# Impact of experimental thermal processing of artificially contaminated pea products on ochratoxin A and phomopsin A

**DOI:** 10.1007/s12550-020-00413-9

**Published:** 2020-10-17

**Authors:** Birgitta Maria Kunz, Alexander Voß, Julia Dalichow, Stefan Weigel, Sascha Rohn, Ronald Maul

**Affiliations:** 1 Department Safety in the Food Chain, German Federal Institute for Risk Assessment (BfR), Max-Dohrn-Str. 8-10, 10589 Berlin, Germany; 2 Hamburg School of Food Science, Institute of Food Chemistry, University of Hamburg, Grindelallee 117, 20146 Hamburg, Germany; 3 Institute for Food and Environmental Research (ILU) e. V., Arthur-Scheunert-Allee 40-41, 14558 Nuthetal, Germany; 4 Present Address: Technische Universität Berlin, Institute of Food Chemistry and Analysis, Gustav-Meyer-Allee 25, 13355 Berlin, Germany; 5 Present Address: Max Rubner Institute, Hermann-Weigmann-Straße 1, 24103 Kiel, Germany

**Keywords:** LC-MS/MS, Ochratoxin A, Phomopsin A, *Pisum sativum*, Bread, Pasta

## Abstract

Fungi of *Aspergillus* and *Penicillium* genus can infect peas (*Pisum sativum*), leading to a contamination with the nephrotoxic and carcinogenic ochratoxin A (OTA). Under unfavourable conditions, a fungus primarily found on lupines, *Diapothe toxica*, may also grow on peas and produce the hepatotoxic phomopsin A (PHOA). To study the effect of processing on OTA and PHOA content, two model products—wheat/rye-mixed bread with pea flour addition and pea pasta—were manufactured at small-business scale from artificially contaminated pea flour. The decrease of OTA and PHOA contents were monitored along the production process as indicators for toxin transformation. Pea bread dough was subjected to proofing for 30–40 min at 32 °C and baked at 250 °C to 230 °C for 40 min. OTA content (LODs < 0.1 μg/kg) showed a reduction in the bread crust (initially 17.0 μg/kg) to 88% and no reduction in the crumb (110%). For PHOA (LODs < 3.6 μg/kg), a decrease to approximately 21% occurred in the bread crust (initially 12.5 μg/kg), whilst for crumb, a less intense decrease to 91% was found. Pea pasta prepared with two toxin levels was extruded at room temperature, dried and cooked for 8 min in boiling water. In pea pasta, OTA was reduced from 29.8 to 13.9 μg/kg by 22% each after cooking, whilst 15% and 10% of the initial toxin amounts were found in the cooking water, respectively. For PHOA, 60% and 78% of initially 14.3 μg/kg and 7.21 μg/kg remained in the cooked pasta. As only the decrease of the initial content was measured and no specific degradation products could be detected, further research is needed to characterise potential transformation products. Heat treatment reduces the initial PHOA content stronger than the OTA content during pasta cooking and bread making. However, significant amounts of both toxins would remain in the final products.

## Introduction

The current popularity of plant-based protein sources makes protein-rich grain legumes and products thereof promising food items for the human diet. Various emerging processed food items are made from legumes, for instance, textured meat substitute burger patties from pea protein or pasta made from pea flour. Legume flour is also used as an additive in bread manufacturing, as mentioned in German customary law (DLMBK 1994). US American federal law also allows the addition of non-wheat flour (CFR 2020a).

**Fig. 1:**
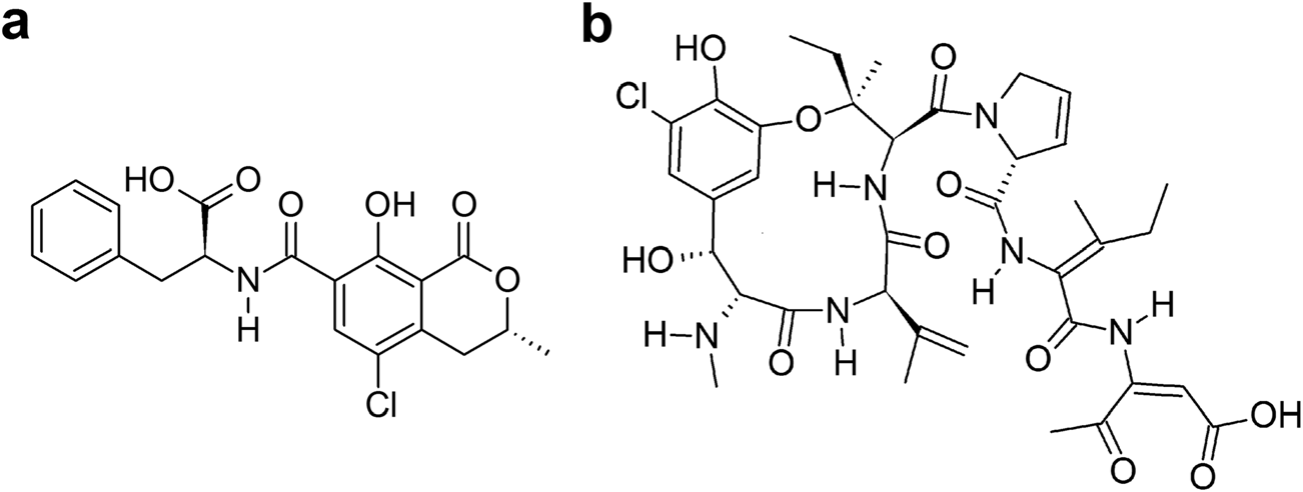
Chemical structures of mycotoxins of **a** ochratoxin A and **b** phomopsin A

Grain legumes have been shown to be occasionally contaminated with mycotoxins. For instance, ochratoxin A (OTA; Fig. 1a) was found in soybeans (*Glycine max*) and products deriving thereof (Ahn et al. 2016; Kolakowski et al. 2016; Warth et al. 2012; Kononenko and Burkin 2008; Beg et al. 2006; Domijan et al. 2005; Valenta et al. 2002; Rafai et al. 2000), beans (*Phaseolus vulgaris*) (BVL 2015; Fakoor Janati et al. 2011; Scudamore et al. 1997; Scott 1997), fermented bean products (Yau et al. 2016) and peas (Kunz et al. 2020). Whilst OTA detection in peas is scarce, the presence of OTA producing fungi, such as *Penicillium* ssp., *Aspergillus ochraceus*, *Aspergillus niger* and *Aspergillus glaucus* (Munimbazi and Bullerman 1996; Hitokoto et al. 1981) has been described for peas.

In field, a known pathogen for lupines (*Lupinus* ssp.) is the fungus *Diaporthe toxica*. It may infect the lupine seeds, producing mycotoxins called phomopsins. One of the major toxins produced is phomopsin A (PHOA; Fig. 1b) (Battilani et al. 2011). After consumption, it binds to tubulin (Battilani et al. 2011), exerting liver toxicity that may lead to a potentially fatal disease called *Lupinosis* in grazing animals, especially sheep (Williamson et al. 1994). However, *D*. *toxica* may not only infect living lupine plants, but acts as a saprophyte as well, growing on already harvested seeds in storage. Incubation experiments simulating unfavourable environmental conditions already showed that the fungus may not only grow and produce phomopsins on lupine seeds during storage, but on other grain legumes as well. Especially on pea seeds, PHOA was formed in high amounts (Schloß et al. 2015a).

Raw grain legumes are frequently subjected to different (food/feed) processing steps as part of value chains. This may include physical, chemical and biological processing of the material. Thus, the mycotoxins contained are affected as well. There are multiple examples of how sorting, soaking and cooking can reduce OTA content in beans (*Phaseolus vulgaris*) (Iha et al. 2009b; Milanez and Leitao 1996; Harwig et al. 1974) and fava beans (*Vicia faba*) (El-Banna and Scott 1984). In addition, more complex processes such as tofu production lead to different distributions of OTA, effectively reducing OTA content in final tofu products (Iha et al. 2009a). Tempeh produced from PHOA contaminated lupines retained most of its toxicity in the nursling rat assay. For its production, the base material had been inoculated with *Rhizopus oligosporus* and immersed in boiling water for 2 min. However, no values on the PHOA reduction were given (Cockrum et al. 1994). The retained observed toxic effect in tempeh seems to be the base for the assumption that phomopsins are stable during cooking as presented by the Australia New Zealand Food Authority ANZFA (2001) whilst stability testing studies of phomopsins under controlled conditions are still lacking.

In wheat bread models, multiple studies on OTA stability were conducted. Some of them did not find any reduction (Osborne et al. 1996; Vidal et al. 2014a, b) or a very small reduction of the OTA content (Scudamore et al. 2003) during bread manufacturing process from flour to bread. Other studies find more pronounced reductions in OTA content. Either they conclude higher OTA reduction during dough proofing than during baking (Milani and Heidari 2017), approximately equal reductions (Valle-Algarra et al. 2009) or a higher reduction during baking than during dough proofing (Bol et al. 2016). One study showed higher OTA reduction in the crust than in the inside of the bread, the crumb (Valle-Algarra et al. 2009). For PHOA in bread, to the authors’ knowledge, no studies have been conducted yet.

Grains such as wheat frequently used for bread preparation are already frequently contaminated with OTA (Schaarschmidt and Fauhl-Hassek 2018). Addition of grain legume flour may contribute to mycotoxin burden in the product. In addition, the aforementioned studies only investigated wheat bread. In Germany, multiple bread varieties with rye as sole grain flour or mixed with other grain flour types are produced, partly with legume flour addition. Due to difference in composition of rye and legume flour, bread with their addition needs adjusted manufacturing parameters. This might lead to differences in the mycotoxin behaviour during manufacturing.

Similar to bread making, several studies report loss of mycotoxins from wheat pasta during cooking. The reduction of deoxynivalenol, a mycotoxin found with high contents in grains, was thoroughly investigated (Bockhorn et al. 2001; Cano-Sancho et al. 2013; de Nijs et al. 2016; Vidal et al. 2016). Mycotoxin levels also decreased during pasta cooking for aflatoxins (Bol et al. 2016), zearalenone (Cano-Sancho et al. 2013; Bol et al. 2016) and enniatins (de Nijs et al. 2016). Ergot alkaloid content was not reduced, but changes in stereochemistry occurred (Tittlemier et al. 2019) and enniatin content reduction was observed during pasta production (Serrano et al. 2016). Specifically, for OTA, three studies showed OTA reduction during cooking (Sakuma et al. 2013; Peng et al. 2015; Bol et al. 2016) and one study did not find a reduction in OTA content in cooked pasta (Vidal et al. 2016). No studies have been conducted on PHOA behaviour in pasta until now. For pea pasta with its altered composition and conditions for preparation (e.g. shorter cooking time), no studies have been conducted yet. It is possible that the alternate properties of pea pasta change the behaviour of the mycotoxins during manufacturing and cooking.

The aim of the present study was to characterise the mycotoxin content along two specific model food products’ processing chains: wheat/rye mix bread with pea flour addition and pasta made from pea flour. It is hypothesized that OTA and PHOA are degraded during the bread manufacturing process. Thus, the decrease of OTA and PHOA contents are monitored as indicators for toxin transformation during processing. The reduction of the mycotoxin content is expected be more pronounced in the bread crust than in the breadcrumb due to exposure to higher temperatures during baking. For pea pasta, as is already shown in the literature for wheat pasta, OTA is expected to leach into the cooking water. PHOA is also expected to migrate into the cooking water.

## Materials and methods

### Chemicals and materials

Ochratoxin A (OTA) certified reference standard solution in ACN (10.05 **±** 0.8 μg/mL from solid standard at 99.5 ± 0.5% , concentration confirmed by HPLC-FLD) was purchased at Romer Labs Division Holding GmbH (Getzersdorf, Austria) and as solid substance (≥ 98%, stored in ACN solution, concentration determined photometrically (ε(333 nm; MeOH) = 6400 L/(mol*cm) according to Cole and Cox (1981))), was purchased from Merck KGaA (Darmstadt, Germany). Phomopsin A (PHOA; ≥ 98%) was purchased from Cayman Chemical Company (Biomol GmbH, Hamburg, Germany). As no extinction coefficient was determined, the solid substance was dissolved in methanol, transferred and dried under nitrogen stream in a pre-weighted vial. The determined weight was solved in a known volume of methanol 6% formic acid (FA) and this concentration was used. d_5_-labelled OTA was purchased from LGC (LGC Standards GmbH, Wesel, Germany).

^15^N_6_-PHOA was isolated from liquid cultures of *D*. *toxica* that only received isotopically labelled nitrogen sources during growth. Details on culture conditions can be found in Schloß et al. (2015b). The mycelium containing culture solution was dried in an infrared rotation vacuum concentrator (RVC 2-33 Infrared rotation vacuum concentrator with condensation trap Alpha 2-4 LD plus, Martin Christ Gefriertrocknungsanlagen GmbH, Osterode am Harz, Germany), milled and extracted with methanol. A portion of this extract was evaporated under a continuous stream of nitrogen and extracted using acetonitrile (ACN)/H_2_O 50/50 (v/v). This extract was subjected to preparative LC under gradient elution with 0.15% aqueous FA and ACN with 0.1% FA (Agilent Technologies 1100 Series, Santa Clara, USA).

In order to test the applicability of the extract as internal standard (IS), an LC-MS/MS measurement of the extract was conducted. No signal of ^14^N_6_-PHOA was found.

ACN and methanol of LC-MS grade as well as FA (ACS reagent, reag. Ph. Eur.) and anhydrous MgSO_4_ (ReagentPlus®) were purchased from Merck KGaA (Darmstadt, Germany). Double deionized water was obtained from a water purification system (Milli-Q® Reference A+ System, Merck KGaA, Darmstadt, Germany).

Fifty kilograms of whole yellow peas (variety ‘Salamanca’) of seed quality was provided by Norddeutsche Pflanzenzucht Hans-Georg Lembke KG, Holtsee, Germany.

### Stock and working standard solution preparation

Stock solutions of standards were acquired as certified reference solutions (OTA for calibration) or as solid substances, either dissolved completely in ACN (for OTA contaminated material spiking) or prepared by weighing and dissolving in methanol with 6% FA (for PHOA). Dilutions of OTA reference standard solution and PHOA stock solution with ACN with 0.1% FA served as calibration stock solutions and as spiking solution. As isotopically labelled mycotoxin internal standard (IS), a solution containing 45 ng/mL d_5_-OTA and approximately 70 ng/mL ^15^N_6_-PHOA mix in ACN with 0.1% FA was prepared.

Several calibration solutions (OTA: 8, PHOA: 7) from 0.19 to 30.7 ng/mL (OTA) and 3 to 26.7 ng/mL (PHOA) were used for quantification.

### Preparation of contaminated pea material

Mycotoxin-free pea flour (blank) was milled, using a centrifugal mill equipped with a 0.5 mm sieve at 18,000 rpm (ZM 200, Retsch GmbH, Haan, Germany). By spiking with OTA standard solution in ACN and adding *D*. *toxica* inoculated pea flour, 1.5 kg pea flour containing 137.4 μg OTA/kg and 132.1 μg PHOA/kg was prepared. This pea flour was used for the bread production. From this pea flour, two further dilutions were made: one at ‘high content’ with 29.8 μg OTA/kg and 14.3 μg PHOA/kg and one at ‘low content’ with 13.9 μg/kg OTA and 7.21 μg/kg PHOA. Those two pea flours were used for pasta production.

### Food processing conditions

#### Bread with pea flour addition

For bread production, sourdough was prepared according to ‘Detmolder one-step-fermentation’ with 2000 g rye flour (type 997), 2000 g water and 80 g starter. The sourdough temperature started at 30 °C and, during fermentation, was kept at 28 °C for 18 h. Three portions of 150 g from each blank pea flour and ‘contaminated bread flour’ were mixed with 816 g wheat flour (type 550), 175 g rye flour (type 997), 350 g sourdough, 29 g block yeast, 23 g salt, 23 g improver (Lezym, CSM Deutschland GmbH, Bingen am Rhein, Germany) and 700 g water at 37 °C, resulting in six doughs of which three were blank and three were contaminated. The dough was mixed in a spiral mixer (Oase-Spiralkneter SK8, Diosna Dierks & Söhne GmbH, Osnabrück, Germany) for 6 min (4 min slow: 690 rpm, 2 min fast: 1360 rpm). The dough, having a temperature of around 28 °C after mixing, was left to rest covered at room temperature for 15 min. Then, each dough was divided into 4 loaves of 550 g, resulting in a total of 24 bread loaves, and the small rest of the dough was put into a plastic freezing bag. Each loaf was moulded by hand. During the manual processing, wheat flour was added as needed by the baker, potentially altering the initial proportions in the recipe. The dough was subjected to proofing for 30–40 min in a fermentation chamber at 32 °C and 75% air humidity (Condac proofer, Condac Kälte und Bäckereitechnik AG, Altstätten, Germany). Finally, after adding a single slit at the top with a knife, the loaves were baked for 40 min at 230 °C in an electric bread oven (Infra AE 508/26, Wachtel GmbH & Co., Hilden, Germany). The initial temperature, 250 °C, was decreased to the baking temperature of 230 °C after volume gain. Following cooling, bread loaves and dough bags were frozen in plastic bags and stored at – 40 °C.

#### Pea pasta

For pasta production, three portions of 485 g from blank pea flour, pea flour at ‘low content’ and ‘high content’ were mixed with 15 g egg powder and 136.5 g water each, resulting in 9 batches of pasta. The mix was homogenised in a kitchen machine (Moulinex Masterchef 570 Duo, Groupe SEB Deutschland GmbH, Frankfurt am Main, Germany) and transferred into a pasta extrusion machine (Nudelmaschine TR 70, Sela-Teigwarengeräte GmbH, Aulendorf, Germany) equipped with a fusilli extrusion mould (N.133, Sela-Teigwarengeräte GmbH, Aulendorf, Germany) and a rotating cutter. Extruded pasta was dried for 4 days in a hot air dryer and stored at room temperature in plastic zip lock bags.

Of all three bags of pasta of each contamination level, two 20 g portions were taken and subjected to cooking, resulting in 18 portions of cooked pasta. In a 600 mL glass beaker covered with a watch glass, 500 mL of deionized water containing 2.5 g NaCl were heated up to boiling point on top of a heatable magnetic stirrer. One portion of pasta was cooked for 8 min and then transferred with a spoon into a 400 mL beaker within 1.5 min. The cooked pasta were further transferred into three 50 mL tubes and put into an infrared rotation vacuum concentrator for 24 h at 10 mbar and 40 °C (RVC 2-33 Infrared rotation vacuum concentrator with condensation trap Alpha 2-4 LD plus, Martin Christ GmbH, Osterode am Harz, Germany). Further, 400 mL beaker, spoon and watch glass were subjected to rinsing with double deionized water and the rinsing solution transferred into the initial 600 mL cooking beaker. The initial cooking beaker was then heat dried at approximately 80 °C for 43 h and the residue weight determined. The dry residue beakers were stored covered at room temperature prior to extraction.

### Sample preparation for analysis

#### Bread with pea addition

A middle strip of 5–6 cm of each loaf was cut with a bread knife and the crust superficially cut from the crumb. One-half of the crumb was diced and dried by infrared rotation vacuum concentrator (24 h). From the crust, the area on both sides of the top slit was used as representative section for further analysis. It was refrained from using the top part in the slit because of its varying width and the shorter contact time with the hot oven air. The bottom part of the crust that was in direct contact with the oven base was omitted as well. From this crust sample, remaining crumb was stripped using the blunt side of the bread knife. Non-baked dough was cut into slices in frozen form and freeze-dried. Dried crumb and dough as well as the crust were stored at room temperature prior to milling.

### Milling

Samples bread crumb, crust, dough, cooked and dried pea pasta and 50 g portions from the pea pasta were ground in a centrifugal mill (0.5 mm distance sieve, 18,000 rpm). Water content was determined for all samples (MA30, Sartorius Lab Instruments GmbH & Co. KG, Goettingen, Germany). For bread, water content ranged from 2.5 to 18.9%. The water content in ‘contaminated pea pasta flour’, pea pasta and cooked and dried pea pasta ranged from 6.6 to 11.0%. Thus, all mycotoxin contents were corrected for water content for comparison and balancing.

### Extraction procedure

The analytical method is based on the draft WI 00275287 of the pre-norm FprN 17279 (DIN 2018), with some modifications as described previously (Kunz et al. 2020). In addition, extraction solvent ratio was modified, a concentration step introduced and the phase separation modified. The resulting method is a simplified QuEChERS approach omitting addition of pre-weighed salts by using saturated aqueous MgSO_4_ solution. Then, 2.5 g of homogenised ground sample were weighed into a 50 mL centrifugation tube and extracted with 2 mL water and 8 mL ACN with 0.1% FA. For each sample except the cooking water residue, three portions were weighed and extracted, resulting in three technical replicates. For the bread dough, crust and crumb, 280 μL of the OTA/PHOA internal standard (IS) were added. After shaking thoroughly for 30 min (Multi Reax, Heidolph Instruments GmbH & Co.KG, Schwabach), samples were centrifuged at 5800×*g* for 30 min at room temperature. Depending on the sample type, between 5 and 7 mL were transferred into 15 mL tubes and dried by infrared rotation vacuum concentrator at 10 mbar and 40 °C for 4 to 5.5 h (RVC 2-33 Infrared rotation vacuum concentrator with condensation trap Alpha 2-4 LD plus, Martin Christ Gefriertrocknungsanlagen GmbH, Osterode am Harz, Germany). Samples were reconstituted with specific volumes (either 700 μL to 1 mL) ACN with 0.1% FA/H_2_O 80/20 (v/v) and shaken for 10 min (Multi Reax, Heidolph Instruments GmbH & Co.KG, Schwabach). To 500 μL of the extract, 100 μL of the OTA/PHOA IS mix was added (or instead 100 μL ACN with 0.1% FA for bread dough, crumb and crust because IS was already previously added). For phase separation, 400 μL of MgSO_4_-saturated water (approximately 330 g anhydrous MgSO_4_ per litre) were added instead of pre-weighed salts. After shaking for 30 s and centrifugation at 17,000×*g* for 10 min at 10 °C, 300 μL of the supernatant were mixed with 300 μL water. Samples were either directly measured or stored at 3 °C for not more than 2 days.

The cooking water residue was extracted in an ultrasonic water bath for 30 min with 10 mL water. After that, 40 mL of ACN with 0.1% FA were added and ultrasonic bath extraction continued for another 10 min. The liquid extract was transferred and centrifuged at 5800×*g* for 10 min. Due to the sodium chloride content in the extract, the water and organic phases readily separated. Of the organic extract, 24.5 mL were evaporated by infrared rotation vacuum concentrator at 10 mbar and 40 °C for 18 h (RVC 2-33 Infrared rotation vacuum concentrator with condensation trap Alpha 2-4 LD plus, Martin Christ GmbH, Osterode am Harz, Germany). From that point, the extraction procedure was equivalent to the formerly described procedure, starting at the reconstitution of the evaporated extract.

The resulting dilution factor from content in the ground sample in μg/kg to the extract solution concentration in ng/mL was 2.9 for cooking water residue whilst for the other samples, depending on the sample intake for analysis, dilution factors of approx. 1.2 resulted.

### Instrumentation and conditions

From the extracts of the three technical replicates (except the cooking water residue, where only one technical replicate was feasible), 4 μL were injected into the LC system (NEXERA X2, Shimadzu Corp., Kyōto, Japan). LC separation was performed using a gradient elution with water (0.1% FA, 300 mg/L ammonium formate) and methanol (0.1% FA, 300 mg/L ammonium formate) and a flow rate of 0.5 mL/min. The gradient program started at 15% organic solvent (B) for 0.8 min, raising to 60% by minute 4.0, to 65% by minute 6.0, to 80% by minute 8.5 and to 95% by minute 11.0. Starting from minute 12.0, the B percentage reverted to the starting conditions of 15% by minute 12.5 and kept until the end of the run at 15 min. A PEEK coated polar C18 analytical column, 100 × 2.1 mm, 5 μm, (ProteCol®, BGB Analytik Vertrieb GmbH, Rheinfelden, Germany) at a column oven temperature of 40 °C was used. MS/MS detection was conducted by a triple quadrupole MS (QTRAP 6500+, Sciex Germany GmbH, Darmstadt, Germany) using both positive and negative electrospray ionisation (ESI) and measuring in multiple reaction mode (MRM). Negative ESI mode was still included because the original method analysed mycotoxins in both positive and negative mode. Curtain gas 40, CAD medium, temperature 300 °C, the +/− ion spray voltage 4500.0 V, GS1 60, GS2 35 and dwell-time varied. MS-parameters for the analytes are presented in Table 1.

**Table 1:** Mass transitions and conditions for LC-MS/MS quantification

Analyte	Retention time [min]	Precursor ion [m/z ]	Measured ion	Product ions [m/z]	Declustering potential (DP) [V]	Collision energy (CE) [eV]	Collision cell exit potential (CXP) [V]
OTA	7.12	404.1	[OTA + H]^+^	239.0	26	31	12
				357.9		19	18
d_5_-OTA	7.08	409.1	[d_5_-OTA + H]^+^	362.8	26	19	18
PHOA	4.11	789.3	[PHOA + H]^+^	323.0	66	35	18
				225.9		49	12
^15^N_6_-PHOA	4.11	795.3	[^15^N_6_-PHOA + H]^+^	227.0	66	49	12

### Validation

Ten blank samples of 2.5 g ground bread crumb, crust and dough, as well as blank pea flour, pea pasta and cooked and dried pea pasta were spiked with 150 μL of an OTA and PHOA mix. This resulted in levels of approximately five times the expected limit of detection (LOD; Table 2). Three additional samples each on two more days were spiked for pea flour. Spiked samples were left to dry overnight. For the cooking water residue, four blank samples were spiked with 300 μL of the same OTA and PHOA mix, also resulting in levels of five times the expected LOD (Table 2). Samples were left to dry openly for approx. 48 h before further sample extraction.

**Table 2:** Method performance of the LC-MS/MS method applied, depending on the different matrices

Matrix	Analyte	LOD [μg/kg]	LOQ [μg/kg]	Intraday precision^a^ [%]	Interday precision^b^ [%]	Lower linear limit [μg/kg]	Upper linear limit [μg/kg]	Correlation coefficient (*r*)	Recovery [%]	Spiked content [μg/kg]
Bread crumb	OTA	0.06	0.19	3.4	–	0.217	35.0	1.000	99.7	0.36
	PHOA	2.79	9.19	9.2	–	3.42	26.74	0.993	136.1	5.70
Bread crust	OTA	0.09	0.31	5.5	–	0.217	35.0	1.000	100.7	0.36
	PHOA	3.57	11.76	11.4	–	3.42	26.74	0.993	140.8	5.70
Bread dough	OTA	0.09	0.30	5.1	–	0.217	35.0	1.000	104.8	0.36
	PHOA	2.42	7.99	8.2	–	3.42	26.74	0.993	132.7	5.70
Pea	OTA	0.07	0.23	5.8	1.1	0.217	35.0	1.000	80.0	0.36
	PHOA	1.68	5.54	5.6	0.7	3.42	26.74	0.996	95.1	5.70
Pea pasta	OTA	0.06	0.21	5.8	–	0.217	35.0	1.000	73.2	0.36
	PHOA	1.57	5.19	5.9	–	3.42	26.74	0.996	85.6	5.70
Cooked and dried pea pasta	OTA	0.07	0.25	7.2	–	0.234	37.8	1.000	68.7	0.36
	PHOA	1.06	3.49	5.7	–	3.69	26.74	0.996	72.6	5.70
Cooking water residue	OTA	0.44 ng^c^	1.46 ng^c^	6.1	–	0.54 ng^c^	87.80 ng^c^	1.000	70.4	1.78 ng^c^
	PHOA	6.82 ng^c^	22.5 ng^c^	3.8	–	8.58 ng^c^	75.33 ng^c^	0.996	83.5	28.5 ng^c^

^a^For intraday precision, 4 samples of cooking water residue and 10 samples of each other matrix were spiked

^b^For interday precision, 3 pea samples on two days and 10 pea samples on the third day were spiked

^c^As the cooking water residue was extracted from the whole pot/beaker, only the amount of total mycotoxin inside the beaker could be determined. Thus, the values are given in ng

Determination of LOD was conducted via blank samples, afterwards the limit of quantification (LOQ) was derived, both according to Wenzl et al. (2016). Repeatability (intraday precision) and recovery were calculated from each set of 10 spiked samples from each matrix or the 4 spiked samples for cooking water residue, respectively. Interday precision was only determined for pea flour, using the average of three spiked samples on 2 days and 10 spiked samples on the third. The linear range was derived from the used calibration concentration range of 7–8 equidistant calibration solutions.

### Data analysis

Data evaluation was performed with MultiQuant Software, V. 3.0.2, AB Sciex Germany GmbH, Darmstadt, Germany.

All values for mycotoxin content in ‘contaminated bread flour’, bread dough, crumb and crust, as well as ‘contaminated pea pasta flour’, pea pasta, cooked and dried pea pasta and cooking residue were corrected by recovery and calculated for the dry mass. The cooking residue was obtained by evaporating the cooking water in the beaker. Thus, it could only be determined as the total amount of OTA and PHOA in ng. For graphic comparison with the pea pasta and the cooked and dried pea pasta, it was calculated back to the relative content in μg/kg in the original pea pasta portion (approx. 20 g) used for the cooking.

Significant difference testing of the sample data was conducted by Student’s *t* test.

## Results

### Method performance

The performance data of the LC-MS/MS method used for quantification of the mycotoxins are given in Table 2. Acceptable sensitivity and linearity for all OTA determinations could be ensured.

### Bread made with contaminated pea flour

During bread making, 150 g of ‘contaminated bread flour’ (the contaminated whole pea flour as previously described) were mixed with the other ingredients to form a total of 2.266 kg initial dough. The initial OTA content in the ‘contaminated bread flour’ used were determined as 157.1 μg/kg by LC-MS/MS. For the initial dough dry content, 17.0 μg/kg OTA are calculated, using the average water contents of the dough samples taken after loaf forming. Those dough samples taken after loaf forming were measured to contain, on average, 17.7 μg/kg OTA for dry mass. However, the difference between bread flour and dough is not statistically significant. For comparison (Table 3), all values were related to the initial mycotoxin content that was calculated for the initial dough mixture. OTA content was significantly reduced (*p* < 0.001) to 88% in the crust in comparison to the initial dough content, whilst no reduction of OTA in the crumb (110%) was observed.

**Table 3:** OTA content measured at the different stages of the bread making process in mixed wheat/rye bread with pea flour addition

Sample	Dough	Loaf	Content [μg/kg]	Content and standard deviation in relation to flour content	Mean over all loaves	Mean over all doughs
Theoretical content in dough calculated from flour content	1–3	1–4	17.0	100 ± 4.7%	100%	100%^a, b^
Dough	1	1–4	18.8	111% ± 2.6%	111%	104%^c, d^
	2	1–4	17.6	104% ± 3.3%	104%	
	3	1–4	16.6	97.5% ± 4.4%	97.5%	
Crumb	1	1	19.8	117% ± 2.6%	116%	110%^a, c^
		2	19.7	116% ± 1.3%		
		3	20.0	117% ± 2.3%		
		4	19.5	116%		
	2	1	18.5	109% ± 3.0%	108%	
		2	18.3	107% ± 1.6%		
		3	18.1	107% ± 2.2%		
		4	18.4	108% ± 2.1%		
	3	1	17.5	103% ± 1.0%	106%	
		2	17.8	104% ± 2.2%		
		3	18.5	109% ± 1.5%		
		4	18.2	107% ± 1.5%		
Crust	1	1	17.1	101% ± 4.0%	96.3%	88.4%^b,d^
		2	16.7	97.9% ± 0.6%		
		3	16.1	94.5% ± 2.7%		
		4	15.7	92.1% ± 1.6%		
	2	1	16.5	97.2% ± 2.0%	88.6%	
		2	14.4	84.6% ± 0.8%		
		3	15.2	89.1% ± 2.5%		
		4	14.2	83.7% ± 1.9%		
	3	1	13.6	79.9% ± 2.1%	80.3%	
		2	13.5	79.6% ± 2.8%		
		3	13.5	79.4% ± 2.1%		
		4	14.0	82.1% ± 2.3%		

^a^Significant difference (*p* < 0.01)

^b^Significant difference (*p* < 0.001)

^c^Significant difference (*p* < 0.01)

^d^Significant difference (*p* < 0.001)

For PHOA, the initial ‘contaminated bread flour’ contained 115.5 μg/kg and the calculated dry content of the initial dough was 12.5 μg/kg, whilst the average of dough samples after loaf forming was measured and determined 14 μg/kg for dry mass (Table 4). PHOA content in the crust was below LOQ. When still estimating the content, PHOA content in the crust was reduced significantly to 20.8% compared to the initial dough content (*p* < 0.001). PHOA in the crumb was reduced to 91%, which is a significant difference to the dough samples after loaf forming (*p* < 0.001).

**Table 4:** PHOA content measured at the different stages of the bread making process in mixed wheat/rye bread with pea flour addition

Sample	Dough	Loaf	Content [μg/kg]	Content and standard deviation in relation to flour content	Mean over all loaves	Mean over all doughs
Content in dough calculated from flour content	1–3	1–4	12.5	100 ± 22%	100%	100%^a^
Dough	1	1–4	13.9	111% ± 5.9%	111%	112%^b, c^
	2	1–4	13.8	110% ± 6.7%	110%	
	3	1–4	14.3	114% ± 5.2%	114%	
Crumb	1	1	9.89	79.0% ± 5.8%	95.9%	90.6%^b^
		2	10.3	82.1% ± 3.5%		
		3	10.3	82.4% ± 5.4%		
		4	17.6	140% ± 18%		
	2	1	10.6	84.3% ± 5.3%	87.5%	
		2	10.4	83.5% ± 3.1%		
		3	13.6	108.5% ± 9.9%		
		4	9.23	﻿72.7% ± 4.0%		
	3	1	11.4	91.5% ± 8.2%	88.4%	
		2	10.2	81.3% ± 4.8%		
		3	10.8	86.1% ± 2.3%		
		4	11.9	94.8% ± 8.0%		
Crust	1	1	2.71	21.6% ± 0.7%	19.3%	20.8%^a, c^
		2	2.41	19.2% ± 1.9%		
		3	2.82	22.6% ± 1.1%		
		4	1.75	14.0% ± 1.4%		
	2	1	4.72	37.7% ± 2.8%	23.4%	
		2	2.08	16.6% ± 1.7%		
		3	2.68	21.4% ± 1.6%		
		4	2.26	18.0% ± 1.3%		
	3	1	1.90	15.2% ± 1.2%	19.7%	
		2	2.83	22.6% ± 4.3%		
		3	2.33	18.6% ± 0.5%		
		4	2.78	22.2% ± 3.2%		

^a^Significant difference (*p* < 0.001)

^b^Significant difference (*p* < 0.001)

^c^Significant difference (*p* < 0.001)

### Pasta made from contaminated pea flour

During pasta manufacturing from the flour and the subsequent drying, no significant reduction in OTA content was observed. Conversely, it seems that the OTA content even slightly increased during pasta manufacturing of the ‘high content’ pasta from 33.2 μg/kg dry mass to 35.6 μg/kg dry mass content (*p* < 0.05) as an average of all three pasta batches.

After cooking, the relative OTA contents in the cooked ‘high and low content’ pea pasta were lower than in the initial dry pea pasta (Table 5). On average, for both ‘high and low content’ pasta batches, 78% OTA remained in both ‘high and low content’ pasta (*p* < 0.001). In the cooking water residues, on average, 12% of the initial amount of OTA was found. For ‘high content’ pasta, this amounted to 15%. For ‘low content’ pasta, the average percentage of the initial amount of OTA in the cooking water residue was 10%. The mass balance for OTA is thus mostly complete but a small gap of 7% (‘high content’) and 12% (‘low content’) remains.

**Table 5:** OTA content measured at the different stages of the pasta making process in pea pasta

Sample	Original content of pea flour (*H* = 29.8 μg/kg; *L* = 13.9 μg/kg)	Dough	Cooking replicate	Content [μg/kg]	Content and standard deviation in relation to pasta content	Mean over all doughs
Pasta	H	1	1–2	39.1	100 ± 3.8%	100%^a^
		2	1–2	36.1	100 ± 2.7%	
		3	1–2	31.6	100 ± 1.9%	
	L	1	1–2	11.8	100 ± 7.9%	100%^b^
		2	1–2	13.2	100 ± 2.2%	
		3	1–2	15.2	100 ± 12%	
Cooked pasta	H	1	1	25.7	65.9 ± 1.1%	78.5% ^a^
			2	32.5	83.2 ± 2.1%	
		2	1	27.7	76.6 ± 0.7%	
			2	26.7	74.0 ± 0.7%	
		3	1	28.4	90.0 ± 0.5%	
			2	25.6	81.0 ± 0.7%	
	L	1	1	8.98	76.2 ± 1.1%	78.0%^b^
			2	9.92	84.2 ± 1.2%	
		2	1	10.7	81.5 ± 2.1%	
			2	11.1	83.9 ± 1.9%	
		3	1	10.4	68.6 ± 1.5%	
			2	11.2	73.9 ± 1.4%	
Cooking water residue	H	1	1	3.97	10.2%	14.5%
			2	6.27	16.0%	
		2	1	9.29	25.7%^c^	
			2	5.39	14.9%	
		3	1	4.96	15.7%	
			2	5.00	15.9%	
	L	1	1	1.10	9.38%	9.89%
			2	0.76	6.43%	
		2	1	1.07	8.14%	
			2	2.01	15.3%	
		3	1	1.77	11.6%	
			2	1.29	8.49%	

^a^Significant difference (*p* < 0.001)

^b^Significant difference (*p* < 0.001)

^c^Treated as outlier and not included in calculation

PHOA content also slightly increased during pasta manufacturing of the ‘high content’ pasta from 16.0 to 19.5 μg/kg dry mass content (*p* < 0.001) as an average of all three pasta batches.

After cooking, 69% PHOA remained in the cooked pea pasta (Table 6). The decrease of PHOA was more pronounced in cooked ‘high content’ than in ‘low content’ pea pasta (60% vs. 78% remaining toxin, respectively; *p* < 0.001). For PHOA, the LOQ in the cooking water residue was at 22.5 μg total PHOA in the beaker and the LOD at 6.82 μg. The amount of PHOA detected for ‘high and low content’ pasta was beneath the LOQ. When estimating the amount of PHOA in the cooking residue as the LOQ, it amounts to 6% (‘high content’) and 13% (‘low content’). A maximum estimation of PHOA in the cooking water residue as the LOQ leads to an overall recovery of initial mycotoxin of 91% in ‘low content’ pasta, thus only a 9% gap. However, the overall recovery for the ‘high content’ pasta with its 66% still implies a fraction of 34% that could not be found in the cooking water.

**Table 6:** PHOA content measured at the different stages of the pasta making process in pea pasta

Sample	Original content of pea flour (*H* =14.3 µg/kg; *L* = 7.21 µg/kg)	Dough	Cooking replicate	Content [μg/kg]	Content and standard deviation in relation to pasta content	Mean over all doughs
Pasta	H	1	1–2	17.7	100 ± 5.3%	100%^a^
		2	1–2	22.8	100 ± 1.0%	
		3	1–2	18.3	100 ± 4.0%	
	L	1	1–2	8.34	100 ± 7.1%	100%^b^
		2	1–2	10.1	100 ± 0.4%	
		3	1–2	8.64	100 ± 4.2%	
Cooked pasta	H	1	1	10.8	61.0 ± 6.4%	60.4%^a^
			2	11.0	62.1 ± 2.2%	
		2	1	10.3	45.3 ± 2.1%	
			2	14.1	61.8 ± 1.8%	
		3	1	12.3	67.4 ± 1.6%	
			2	11.9	65.1 ± 1.6%	
	L	1	1	7.73	92.6 ± 3.7%	78.3%^b^
			2	7.06	84.6 ± 3.8%	
		2	1	6.77	67.4 ± 1.1%	
			2	7.56	75.2 ± 1.9%	
		3	1	6.13	70.9 ± 3.4%	
			2	6.82	78.9 ± 5.0%	
Cooking water residue	H	1	1	1.11	6.29%	5.80%
			2	1.11	6.29%	
		2	1	1.12	4.89%	
			2	1.13	4.94%	
		3	1	1.13	6.20%	
			2	1.13	6.17%	
	L	1	1	1.12	13.4%	12.5%
			2	1.13	13.5%	
		2	1	1.13	11.3%	
			2	1.13	11.3%	
		3	1	1.11	12.9%	
			2	1.11	12.9%	

^a^Significant difference (*p* < 0.001)

^b^Significant difference (*p* < 0.001)

## Discussion

### Method performance

For PHOA analysis, LOD and LOQ were close to the maximum level given by the Australian and New Zealand governments for phomopsins of 5 μg/kg in lupine seeds (ANZFSC 2017). The repeatability was good for all analytes and matrices. The recoveries obtained for pea flour, pea pasta, cooked and dried pea pasta and cooking water residue (68.7–80% OTA and 72.6–95.1% PHOA) differ from the recoveries of bread with pea flour addition dough, crust and crumb (99.7–104.8% OTA and 132.7–140.8% PHOA). This might be attributed to the differences in the extraction method for samples analysed in the present study. For pea flour, pea pasta, cooked pea pasta and the cooking water residue, the IS was added after an initial extraction and concentration step, before phase separation. For bread dough, bread crumb and bread crust, the IS was added right at the first extraction step. In addition, the validation of those two matrix groups were conducted at different dates and with different spiking mixes. The recovery of phomopsin A for bread with pea flour addition is unusually high. This cannot be attributed to carryover in the system as during validation, blank samples of bread crust, crumb and dough that had not been spiked did not show PHOA signals (retention time 4.14 min and Qualifier/Quantifier ratio 0.88).

Multiple studies investigate the effect of processing on mycotoxin content along the value chain from raw materials to the final products. However, there is little data specifically on grain legumes. As grains and grain products are the world’s most consumed food items, most studies were conducted on grains. Schaarschmidt and Fauhl-Hassek (2018) reviewed comprehensively the effect of processing steps on the mycotoxin content for grains.

### Bread making

#### Ochratoxin A

For bread, many processing studies have been conducted on OTA alone (Osborne et al. 1996; Scudamore et al. 2003; Valle-Algarra et al. 2009; Vidal et al. 2014a, b; Bol et al. 2016; Milani and Heidari 2017). Wheat flour is the bulk material for most bread varieties and is itself frequently contaminated with OTA (Schaarschmidt and Fauhl-Hassek 2018). Rye flour, which is also a common bulk ingredient for bread baking in Germany, has also been shown to contain OTA (Kolakowski et al. 2016).

Some studies found no significant OTA reduction (Osborne et al. 1996; Vidal et al. 2014a, b) or considered the effect small (Scudamore et al. 2003). These studies investigated breads with loaf sizes between 260 and 800 g, conforming the US federal law as well as German customary law definitions that bread loaves have to weigh at least 250 g (CFR 2020b; DLMBK 1994). In the present study, OTA reduction (12%) has only been found in the bread crust. The crust makes up only a small part of the total bread mass. Consequently, if extrapolated to the whole bread loaf, the reduction in OTA content would be either very small or not observable. As the three aforementioned studies only investigate the whole bread, the lack of OTA reduction as reported in those studies could be attributed to this effect, whilst in our investigation on both crust and crumb, the reduction in the crust becomes visible.

The three following studies depict significant reduction in OTA content during baking. They used much lower loaf weights, meaning smaller loaves, higher relative surface area and more crust compared to crumb were present: 33% reduction with a loaf weight of 80 g (Valle-Algarra et al. 2009), 54% reduction with 70 g (Bol et al. 2016) and 5–26% reduction between the dough before and after baking with 50 g (Milani and Heidari 2017). Those small bakery goods are too small to be compared to the true breads prepared for the present study. The higher exposure to the oven heat of the small loaves might explain why a reduction is observable in the first place. For the present study, only crust and crumb were investigated, not the whole bread. Thus, the values are not directly comparable. The only OTA reduction observable in the present study was in the crust with 12% OTA reduction. This is well below the reduction of 54% in the whole bread in Bol et al. (2016)’s study that includes fermentation steps to the process. Even though this leads to the assumption that fermentation played a bigger role there, they only found 7% OTA reduction during fermentation alone. When focussing on the baking step only, Milani and Heidari (2017) found reduction values in the whole bread with 5–26%. Even though not directly comparable, this is similar to the 12% reduction in the crust in the present study. Valle-Algarra et al. (2009)’s OTA reduction during baking in the whole bread is higher than in the present study with, on average, 33%.

Valle-Algarra et al. (2009) also studied bread crust and crumb separately and showed a reduction of OTA content in the crust (20–51%). However, the crust value given was calculated from flour to baked bread and does still include possible reductions from fermentation steps. The 12% OTA reduction in the crust in the present study is smaller than the results described. Converse to the lack of OTA reduction in the crumb in the present study, Valle-Algarra et al. (2009) also observe OTA reduction in the crumb (7–38%). The observation that OTA reduction was more pronounced in the crust is still common in both the former study and the present study. Milani and Heidari (2017) tested different yeast varieties and sourdough applications, achieving significant OTA reduction rates during dough proofing of 19 to 83% with two proofing periods of 90 min at 30 °C and another 10 min follow-up. Other reduction rates observed during proofing were between 30 and 34%, but incubating for 1 h at 30 °C (Valle-Algarra et al. 2009) and 7% (Bol et al. 2016). In the present study, dough samples were taken after a first short dough rest step at room temperature. The proofing step at 32 °C for 30–40 min followed before baking. Thus, microbial processes might have played a role in reductions from dough to baked bread, especially during this period in the fermentation chamber where optimal conditions for the yeasts are given. This is not relevant for OTA in the present study, as a measurable OTA reduction in the crumb is missing.

In the oven, the crust is exposed to outer temperatures of up to 230–250 °C. In a model system testing pure OTA without solvent, a temperature of 120 °C already led to transformation and, consequently, reduction of OTA content (Sueck et al. 2019). The temperature of 230–250 °C in bread baking is also comparable to the environment or final bean temperature during coffee roasting. Multiple studies have already shown a reduction of OTA during coffee roasting (La Pera et al. 2008; Nehad et al. 2005; Perez De Obanos et al. 2005; Romani et al. 2003; van der Stegen et al. 2001). The reduction may partially be attributed to chemical transformation, such as binding to polysaccharides (Bittner et al. 2013), transformation to the isomer 2′R-OTA, decarboxylation of OTA to decarboxy-OTA, as well as cleaving to the phenylalanine-deficit OTα and OTα amide (Cramer et al. 2008; Bittner et al. 2015). It is possible that this kind of transformation is also partially the cause for the OTA reduction in the bread crust in the present study. Identification of 2′R-OTA in malt bread market sample supports this assumption (Sueck et al. 2019). Due to the lack of a quantitative method, the OTA degradation products OTα, OTα amide and 2′R-OTA were monitored only qualitatively according to the MS parameters as published by Paoloni et al. (2018), Bittner et al. (2013) and Sueck et al. (2019). No intense LC-MS signals were detectable in the present study, only small signals that suggest the presence of 2′R-OTA in several visually dark crust samples.

#### Phomopsin A

**Fig. 2:**
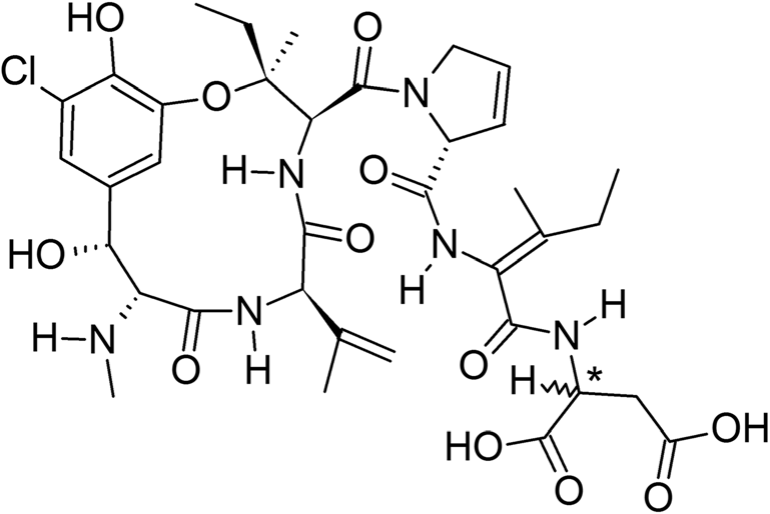
Chemical structure of Phomopsin D

Cockrum et al. (1994) already identified and characterized the PHOA derivative phomopsin D (Fig. 2) as a possible product of treatment with *Rhizopus oligosporus* during tempeh production from lupines. It is possible that a chemical reduction or similar transformation processes have occurred in the bread dough in the present study. Especially the small decrease between PHOA content in the dough samples before proofing and the crumb (21%) might be partially attributed to enzymatic microbiological transformation processes from both yeast and sour dough. Cockrum et al. (1994) also submerged the final tempeh for 2 min in boiling water. Because there is no data on the PHOA content before and after this cooking step, it is not clear whether it might have led to heat induced transformation products or leaching into the cooking water. However, in the present study, the reduction from dough before proofing and crust (91% relative to calculated initial flour PHOA content) is much higher than the reduction in the crumb. No literature data on the effect of baking and thus dry heating on PHOA is available. Still, a form of thermally induced binding or transformation of PHOA is likely an explanation of the PHOA reduction in the bread crust.

Other than enzymatic or thermal reduction, other ways of elimination of PHOA might play a role. Incubation at ruminant body temperature of 39 °C in rumen extract diluted with artificial saliva fluid at pH 6.5–7 already led to 55% transformation of PHOA or 51% if the microflora was removed by centrifugation prior to incubation (Vogel 1988). The elimination was considered largely non-enzymatic. It is possible that those unspecified elimination processes that do not rely on enzymatic activity or high temperatures have contributed to PHOA elimination in the bread, for example by binding to matrix components. Vogel (1988) also describes incidents of elimination of PHOA in alkaline buffer solutions, pH 8.5, that were frozen and thawed in storage for 1 week (60%), suggesting that PHOA may be eliminated even under relatively mild conditions. As PHOA possesses a peptidic structure, strongly alkaline conditions in general may lead to its elimination (Battilani et al. 2011). Additionally, during evaporation of standard solutions at 50 °C with acid present, 20–80% of the standard in stock solutions were eliminated (Vogel 1988).

On the German market, there are already multiple baking goods containing legume flour. Besides pea flour, lupine flour may be used as addition. As phomopsins naturally occur in lupines, the collected data may also help to extrapolate data for breads with lupine flour addition.

### Processing of pea pasta

#### Ochratoxin A

In contrast to the expectations, OTA content slightly increased during the pasta production step from flour to dry pasta from 33.2 μg/kg dry mass to 35.6 μg/kg dry mass content as an average of all three pasta batches of ‘high content’ flour (*p* < 0.05). This might be the effect of inhomogeneity of the material.

Four different studies described the effect of cooking on the OTA content in pasta: Sakuma et al. (2013) produced fettuccine from spiked durum wheat (*Triticum durum*) semolina dough. The remaining amounts of OTA in cooked pasta from two differently contaminated doughs (5 μg/kg and 10 μg/kg) were both at 66% of the initial level. In the cooking broth, 33% and 35% were found, effectively completing the mass balance. The authors suggest that OTA reduction can be attributed solely to leaching into the cooking water. Bol et al. (2016) observed a similar reduction of OTA content to 65% in fettucine. It was expressed as the ratio of original flour dry content compared to final pasta dry content and thus not adjusted to dilution by other ingredients. Conversely, only 8% were found in the cooking water. Peng et al. (2015) prepared wheat pasta strips from contaminated wheat flour with added sodium carbonate. Reduction by 23% for the dry material was achieved from flour to cooked pasta at two original flour contents (42.9 μg/kg and 138.3 μg/kg). However, reduction was more pronounced in the steps from flour over dough to fresh pasta, where already 13% to 20% OTA reduction were achieved. The authors suggest this pronounced reduction may be attributed to the alkaline environment in the dough mixture (pH 8.14–9.20). Only observing the step from fresh pasta to cooked pasta, OTA content was reduced to 98% and 88%. Vidal et al. (2016) found no significant reduction of the mycotoxin content during cooking of durum spaghetti with or without egg added as further ingredient. However, with increasing cooking time, OTA content increased in the cooking water, suggesting a certain leaching rate of OTA.

Compared to Sakuma et al. (2013)’s results, the present study shows a slightly lower OTA reduction in the pasta (66% vs. 78%). Both studies used spiked material but Sakuma et al. (2013) use base material of a lower OTA content (5 μg/kg and 10 μg/kg vs. 13.9 μg/kg and 29.8 μg/kg in the present study). Both find the mass balance of OTA in pasta and cooking water complete, suggesting the way of OTA elimination is leaching into the cooking water. Different pasta shapes and thus relative surface area (fusilli that are extruded with a middle spine and long and flat fettuccine), cooking time, pasta/cooking water ratio and the ingredients can influence the retention of OTA. Bol et al. (2016)’s results show an OTA dry mass retention of 65% in the cooked pasta as compared to the original spiked flour, thus similar results as Sakuma et al. (2013). In the cooking water of Bol et al. (2016)’s experiments, only 8% OTA were found, leaving a gap in the mass balance. Even though the ratio pasta/cooking water was lower with 1/7 as compared to 1/25 in the present study, Bol et al. (2016) used a much longer cooking time (15 min as compared to 8 min). Given the more accessible shape of the pasta and the lacking indication of the authors that a drying step was included, this indicated that the fresh pasta was cooked much longer than needed for an *al dente* state. For the investigation of the OTA content in cooking water, no information on the cooking water sampling is given. In the present study, the cooking water including its cloudy pasta debris content were evaporated to dryness and then this cooking water residue extracted. If the cooking water including debris was filtered and only the water investigated, the assumption is that the OTA migration into the cooking water would be lower than the actual reduction achieved. Thus, the approach in the present study to investigate the whole cooking residue and not only the filtered cooking water might be a possible explanation for the discrepancy. Both Peng et al. (2015) and Vidal et al. (2016) used inoculated base material for their studies as opposed to the spiking approach in the present study. They found either lower OTA reduction from dry pasta to cooked pasta of 98% and 88% or no significant reduction in OTA content. Furthermore, both studies have pasta/cooking water ratios from 1/4 to 1/5 as compared to the ratio of 1/25 in the present study, giving another possible explanation for lower OTA reduction by leaching into the cooking water.

#### Phomopsin A

PHOA content slightly increased during the pasta production step from flour to dry pasta from 16.0 to 19.5 μg/kg dry mass content as an average of all three pasta batches of ‘high content’ flour (*p* < 0.001). Changes to the matrix integrity might have led to a better accessibility of PHOA during extraction. PHOA was introduced to the contaminated pea flour batches as flour from *D*. *toxica* inoculated peas. As naturally contaminated peas were used, a complex mixture of initial pea material and hyphae of the fungus *Diaporthe toxica* was present. Mechanical mixing, soaking of the mixture in water and the extrusion process might have further destroyed the hyphae structures and made PHOA more accessible for extraction.

The PHOA mass balance data of ‘high content’ pasta show a gap of 34%. This suggests another way of elimination than leaching into the cooking water, indicating transformation (either in the pasta itself or in the cooking water) or matrix binding. As described before, it is possible that the heat and cooking had an impact on the transformation process (Cockrum et al. 1994). Other non-enzymatic and non-thermal elimination processes might have also played a role (Vogel 1988).

The results already show reductions during the production and preparation process of the two pea products: pea pasta and wheat/rye mix bread with pea flour addition. Both products show similar behaviour in OTA reduction like some previously published studies on grain products. For bread production, the loaf size and the focus on the whole bread or the crust and crumb specifically seem to be the most significant factors. For pasta, the way of introduction of OTA into the material, the pasta/cooking water ratio, the cooking time as well as the different composition of pea pasta and durum pasta all seem to play a role.

OTA reduction in both, pea pasta and wheat/rye mix bread with pea flour addition, is lower than PHOA reduction. However, the method of introduction of both OTA and PHOA was fundamentally different as OTA was spiked and PHOA introduced by using pea material artificially contaminated with *D*. *toxica*. Thus, a direct comparison for the two toxin contents in not feasible. It is still possible that PHOA is less heat stable than OTA. However, PHOA has already shown tendencies to be eliminated non-enzymatically and non-thermally from biological systems (Vogel 1988). To get a deeper insight into those processes, further research is needed.

As leaching of OTA into water seems to eliminate a certain amount of it from grain legumes (Iha et al. 2009b; Milanez and Leitao 1996; Harwig et al. 1974; El-Banna and Scott 1984), washing, soaking and/or cooking and discarding the excess water may lead to a reduction of OTA content in grain legume products. Roasting at high temperatures also possibly leads to OTA reduction as many studies on coffee roasting show (La Pera et al. 2008; Nehad et al. 2005; Perez De Obanos et al. 2005; Romani et al. 2003; van der Stegen et al. 2001). Similarly, washing, soaking and cooking of grain legumes might lead to PHOA reduction in grain legume products as this study shows a migration of PHOA from pea pasta into the cooking water. It is unclear how large the role of thermal processes in PHOA reduction is. The higher reduction of PHOA content in the bread crust compared to the crumb in the present study suggests a reduction effect at the bread surface exposed to high oven temperatures (230–250 °C). However, none of those processes leads to complete elimination of OTA or PHOA from the products. Instead, appropriate storage management, such as dry and cool conditions, may be a more promising approach to avoid the production of OTA and PHOA during storage altogether.

Findings of OTA (Kunz et al. 2020) and the presence of potentially OTA producing fungi on peas (Munimbazi and Bullerman 1996; Hitokoto et al. 1981) and that PHOA is produced on peas once infected in storage (Schloß et al. 2015a) indicate that a health risk for consumers might be possible. The observed reductions in wheat/rye mix bread and pea pasta may help future risk assessment through derivation of process factors. More research on possible transformation products would complement the risk assessment even further.

## Abbreviations

PHOA: Phomopsin A
OTA: Ochratoxin A
IS: Internal standard
LOD: Limit of detection
LOQ: Limit of quantification
